# Contribution of Metabolomics to the Understanding of NAFLD and NASH Syndromes: A Systematic Review

**DOI:** 10.3390/metabo11100694

**Published:** 2021-10-11

**Authors:** Cristina Piras, Antonio Noto, Luciano Ibba, Martino Deidda, Vassilios Fanos, Sandro Muntoni, Vera Piera Leoni, Luigi Atzori

**Affiliations:** 1 Department of Biomedical Sciences, University of Cagliari, 09042 Monserrato, Italy; cristina.piras@unica.it (C.P.); lucianoibba96@gmail.com (L.I.); smuntoni@unica.it (S.M.); vleoni@unica.it (V.P.L.); latzori@unica.it (L.A.); 2 Department of Medical Sciences and Public Health, University of Cagliari, 09042 Monserrato, Italy; martino.deidda@tiscali.it; 3 Neonatal Intensive Care Unit, Department of Surgical Sciences, University of Cagliari, 09042 Monserrato, Italy; vafanos@tiscali.it

**Keywords:** metabolic syndrome, non-alcoholic fatty liver disease, non-alcoholic steatohepatitis, metabolomics

## Abstract

Several differential panels of metabolites have been associated with the presence of metabolic syndrome and its related conditions, namely non-alcoholic fatty liver disease (NAFLD) and non-alcoholic steatohepatitis (NASH). This study aimed to perform a systematic review to summarize the most recent finding in terms of circulating biomarkers following NAFLD/NASH syndromes. Hence, the research was focused on NAFLD/NASH studies analysed by metabolomics approaches. Following Preferred Reporting Items for Systematic Reviews and Meta-analysis (PRISMA) guidelines, a systematic search was conducted on the PubMed database. The inclusion criteria were (i) publication date between 2010 and 2021, (ii) presence of the combination of terms: metabolomics and NAFLD/NASH, and (iii) published in a scholarly peer-reviewed journal. Studies were excluded from the review if they were (i) single-case studies, (ii) unpublished thesis and dissertation studies, and (iii) not published in a peer-reviewed journal. Following these procedures, 10 eligible studies among 93 were taken into consideration. The metabolisms of amino acids, fatty acid, and vitamins were significantly different in patients affected by NAFLD and NASH compared to healthy controls. These findings suggest that low weight metabolites are an important indicator for NAFLD/NASH syndrome and there is a strong overlap between NAFLD/NASH and the metabolic syndrome. These findings may lead to new perspectives in early diagnosis, identification of novel biomarkers, and providing novel targets for pharmacological interventions.

## 1. Introduction

### 1.1. MetS General Characteristics

Metabolic syndrome (MetS) is a global epidemic that is leading to an increased risk of developing chronic diseases such as type 2 diabetes and cardiovascular diseases. The exact cause of MetS is not known, however the pathogenesis seems to be due to the presence of multiple factors including genetic predisposition, life-style habits, epigenetic modifications, and environmental expositions. Recent studies suggest that among risk factors, central obesity, hypertension, glucose intolerance, hypertriglyceridemia, and low serum high-density lipoprotein (HDL) have been considered important for the development and progression of this syndrome [1]. Awareness about the existence of the metabolic syndrome is relatively recent, since it was officially recognized by the World Health Organization (WHO) twenty-tree years ago (1998), when Alberti et al. [2] described this condition identifying as a mandatory criterion the presence of insulin resistance. The occurrence of at least two additional risk factors among obesity, dyslipidaemia, hypertension, and microalbuminuria [3] was also needed to attest the presence of MetS. In 2005, a new definition of MetS was shaped by the National Cholesterol Education Program (NCEP), the Adult Treatment Panel III (ATP III), the American Heart Association, and the National Heart Lung and Blood Institute [4]. MetS is present if three or more among the following five criteria are met: waist circumference over 101 cm (men) or 90 cm (women), blood pressure over 130/85 mmHg, fasting triglyceride (TG) level over 150 mg/dL, fasting high-density lipoprotein (HDL) cholesterol level less than 40 mg/dL (men) or 50 mg/dL (women), and fasting blood sugar over 100 mg/dL [5]. 

### 1.2. NAFLD and NASH

Accumulating evidence supports a metabolic association between MetS and non-alcoholic fatty liver disease (NAFLD) [6,7]. Their pathogenesis seems to have common pathophysiological mechanisms which involve cellular metabolism and has insulin resistance as a key factor. NAFLD represents a group of disorders that have in common the presence of fatty liver disease in individuals who do not consume alcohol or who consume very small quantities (less than 20 g of ethanol per week). NAFLD is considered the hepatic expression of MetS, with which it shares aetiology, prognosis, and treatment. It is characterized by an excessive accumulation of toxic lipids in the liver, including triglycerides, FFA, ceramides, and free cholesterol. NAFLD has become the most common cause of chronic liver disease in the United States, affecting over 3–5% of the population [8]. NAFLD may progress to an advanced stage named NASH (about 20–30% of individuals with NAFLD), in which the presence of inflammation, fibrosis, and cell damage can lead to cirrhosis and hepatocellular carcinoma (about 15% and 4–27% of individuals with NASH, respectively). However, it should be noted that NASH could progress to hepatocellular carcinoma in the absence of signs of cirrhosis. Currently, diagnosis is performed measuring the activity of hepatic enzymes, by examining the liver with ultrasound, computed tomography, and nuclear magnetic resonance spectroscopy. However, a clear correlation between the enzymatic alterations and the extent of steatosis do not exist, not even when is detected on liver biopsy. Liver biopsy is still considered the most informative examination, but, as it is not usually performed in subjects without alterations of the hepatic enzymes, the true prevalence and distribution of NAFLD is underestimated. Over the last few years, omics technologies have allowed us to obtain an integrated view of the NAFLD/NASH phenotype. The application of genome-wide associated study allowed the identification of several SNPs associated with MetS and NAFLD. Among these, the nonsynonymous rs738409 C/G variant in PNPLA3 (patatin-like phospholipase domain containing 3), which encodes the amino acid substitution I148M, was found as the major genetic component of NAFLD and NASH. The rs738409 was significantly associated with the accumulation of fat in the liver and with the histological disease severity and progression of NAFLD [9]. For this reason, it was considered a promising therapeutic target. However, its translational value from the bench to the bedside has been limited, as the reproducibility of these SNPs differed among groups of patients [10]. A step forward over the genomic era may be represented by the analysis of small metabolites and lipids contained in biofluids (plasma, serum, urine, and saliva). This approach is named metabolomics, and it may be particularly useful for the study of chronic metabolic diseases in which the phenotype is complex and dynamic, resulting from the occurrence of multiple interactions among genetic and environmental factors. For these reasons, metabolomics is expected to provide many more additional insights and clues to the mechanisms of biological processes and functions, and thus may increase our knowledge of the development and progression of the disease such as NAFLD and NASH. Therefore, this work aimed to conduct a systematic review of human studies on metabolite markers of MetS and to provide a list of shared metabolic pathways.

## 2. Methods

This systematic review follows the PRISMA guidelines and is reported in accordance with the PRISMA statement (http://www.prisma-statement.org/ accessed on 16 February 2021).

### 2.1. Search Strategy

A systematic search was conducted on PubMed for all publications with relation to metabolomics biomarkers of Metabolic syndrome reported from 2010 to July 2021, using the following combinations of terms: “Metabolomics or Metabonomics” and “Metabolic Syndrome” and “Human” and “Liver not Review” and “NAFLD/NASH”. Initially, titles and abstracts of all identified studies were screened and reviewed based on the established selection criteria. 

### 2.2. Selection Criteria 

English articles were selected based on their titles and abstracts for full-text review according to their relevance to the issue of interest. The following inclusion criteria were applied with no restriction to the bio-specimen used: identification of specific metabolites in NAFLD/NASH or Metabolic syndrome; identification of potential biomarkers of NAFLD/NASH or Metabolic syndrome diagnosis, or with a diagnosis of one or more traits of the disease; and level of standardization of the analytical platforms used and their limitations. Only metabolomics studies were included. Other “omics” results were excluded. In addition, reviews and studies made on animal models of Metabolic syndrome or on cell model systems were excluded. Systematic reviews and meta-analyses were excluded from the research.

### 2.3. Data Extraction

The selected studies were thoroughly examined, and the following information was extracted from each article: name of first author, year of publication, sample size, analytical platform used, use case, relevant biomarkers candidates, validation of biomarkers, statistical details, and relevant comments about the study. Data were independently extracted by three different reviewers (C.P, A.N., and L.I.) and disagreements regarding the selected information were solved by further review and discussion among them.

## 3. Results

**Figure 1:**
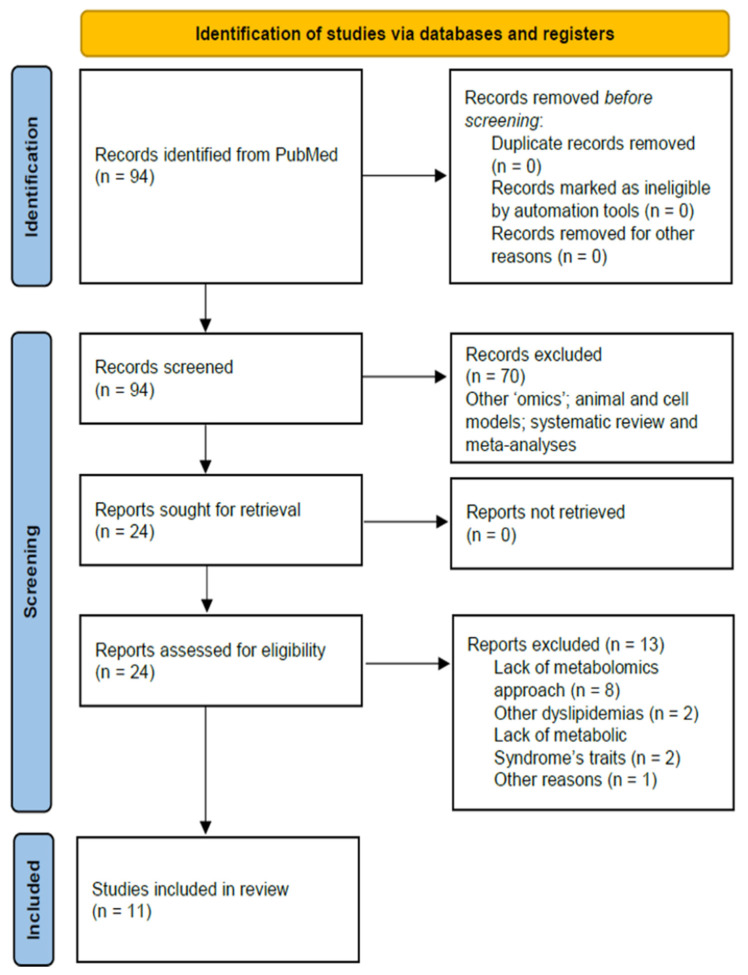
Flowchart of study selection. These articles were published between 2010 and 2021, the diagram shows the method by which relevant studies were retrieved from the databases, assessed, and selected, or excluded.

A total of 94 studies were evaluated following the literature search (Figure 1). 

The full text was revised for 24 articles, and 13 were excluded from analysis after reading the full text (Table 1). The remaining 11 were considered relevant for inclusion in the systematic review. Therefore, we provided a narrative synthesis of the main results, organized by specific biochemical metabolism. As a second level, we organized results by the type of molecule identified.

### 3.1. NAFLD/NASH Biomarkers: Results from Case/Control Studies

Most of the studies were performed in populations aged between 7 and 80 years. Generally, NAFLD/NASH cases were compared with healthy controls (see Table 2). Biomarkers were mostly identified using targeted MS metabolomics or lipidomics on blood, urine, and saliva. About 100 different metabolites were identified and are presented in Table 2 with associated references and classified by families and direction of variation, as well as analytical methods for metabolomics/lipidomics and used statistical parameters/cofactors. The main classes were amino acids and derivatives, carbohydrates and derivatives, glycolysis related metabolites, glycerophospholipids, glycerolipids, sphingolipids, fatty acids, cholesterol and oxysterols, steroids, and peptides.

### 3.2. Metabolism of Amino Acids and Derivatives

Three studies showed an increase in the concentration of alanine. Two of these studies, performed by Männisto et al. [11], and by Stechemesser et al. [12], showed an increase in alanine concentration in patients with MetS and NASH compared to healthy controls and with subjects with NAFLD. In particular, Stechemesser et al. [12] showed that the concentration of alanine was very different between the healthy control group and the MetS group, but did not discriminate when compared to subjects with high and normal iron parameters. The third study by Sookoian et al. [13] found an increased ratio of alanine/pyruvate in NAFLD patients compared to healthy controls. In particular, it was shown that hepatic transcriptional activity of aminotransferases (AST and ALT) was significantly upregulated in NAFLD patients and alanine was correlated with hepatic mRNA expression of GPT (gene encoding the cytosolic isoform ALT1), GPT2 (gene encoding the mitochondrial isoform ALT2), and GOT1 (gene encoding the cytoplasmic GOT). Furthermore, the L-alanine:pyruvate ratio was significantly correlated with BMI (R = 0.37, *p* = 0.01) even after adjustment for HOMA-IR and aminotransferase concentrations (β ± SE: 0.42 ± 0.18; *p* = 0.02). Six studies by Bhupathiraju et al. [14], Feldman et al. [15], Männisto et al. [11], Stechemesser et al. [12], Troisi et al. [16], and Masarone et al. [17] showed an increased concentration of branched-chain amino acids (BCAAs) in obese and NASH patients. Bhupathiraju et al. [14], while investigating the metabolomic profiles associated with distinct dietary patterns among a sample of Asian Indians living in the United States, found that western/nonvegetarian dietary pattern was positively associated with a high concentration of BCAAs.

Apparently, in contrast with the previous author, Feldam et al. [15] found an inverse correlation between obese subjects and BCAAs profile. However, the authors pointed out that obese patients had healthy livers, thus suggesting that obese patients with healthy liver have low blood concentrations of BCAAs. In agreement with Bhupathiraju et al. [14], the study performed by Mannisto et al. [11] discovered that the levels of serum BCAA were higher in NASH patients compared to patients with simple steatosis. The same variation in the concentration of BCAAs was observed by Stechemesser et al. [12] in MetS patients compared to healthy subjects. A significant increase in the BCAA isoleucine was also observed by Troisi et al. [16] in the saliva collected from paediatric obese patients compared to normal-weight subjects. Masarone et al. [17], in a recent study from 2021, showed how the BCAAs increased with the progress of the disease severity from steatosis disease to NASH, and NASH-cirrhosis. As for BCAA, the amino acid glutamate was found significantly increased in NAFLD patients by Lovric et al. [18] and Stechemesser et al. [12], whereas Sookoian et al. [13] were the only ones to identify an increased concentration of the amino acid kynurenine associated with serum concentrations of aminotransaminases and with BMI and HOMA-IR. 

### 3.3. Metabolism of Fatty Acids and Derivatives 

Lovric et al. [17] identified an increase in the concentration of sphingomyelin (SM) at the level of the ectopic adipose tissue. From the SM pool, diSM (18:0) stranded out with the highest positive correlation towards ectopic fat depots. A group of unsaturated fatty acids correlated with the metabolic status of obese individuals was found by Ni et al. [19]. The metabolites dihomo-gamma-linolenic acid (DGLA) and palmitoleic acid resulted significantly elevated in overweight/obese subjects with diabetes compared to their healthy counterparts. According to the authors, these metabolites were also able to predict the future development of MetS. In contrast to the previous authors, both Zhou et al. [9] and Feldman et al. [15] found a decreased concentration of lipids in NASH/NAFLD patients. In particular, the study of Zhou et al. 2016 [20] reported a reduction in the concentration of lysophosphocholine (16:0) in subjects with NASH compared to subjects without NASH, while Feldman et al. [15] found that low concentrations of some PCs (acyl–alkyl PCs) and sphingolipids were characteristic of obese NAFLD patients compared to healthy obese or lean subject.

### 3.4. Metabolism of Vitamin and Ketone Bodies 

The alteration of retinoids was revealed by Zhong et al. [21]. The authors highlighted a reduction in the concentration of all-trans-retinyl palmitate-d4 (RP), all-trans-retinoic acid (atRA), 4-oxo- atRA, and 13-cisRA in subjects with NASH compared to controls. The NASH group showed also significant reduction in the ketone bodies β-hydroxybutyrate (β-OHB) (*p*-value = 0.004) and acetoacetate (*p*-value = 0.018) as demonstrated by Mannisto et al. [11]. In particular, lower levels of β-OHB were associated with the NASH predicting score *p* = 0.001.

## 4. Discussion

Cellular metabolism plays a crucial role in both health and disease, mirroring interactions between the host genome and the environment. Environmental and lifestyle factors have the potential to alter the individual metabolic phenotype both directly, by inducing perturbations in various metabolic pathways, and indirectly, by promoting epigenetic changes, which in turn lead to changes in gene expression, transcripts, and ultimately in the metabolic profile of a given cell, tissue, or biological fluid [22]. In the present systematic review, 10 studies matching with certain inclusive criteria were analysed, aiming to highlight common potential metabolites that characterized the onset or progression of the MetS, with a particular focus on metabolic abnormalities in the liver such as NAFLD and NASH syndromes. The discovery of new biomarkers in the study of the NAFLD/NASH could offer new investigative tools allowing an early diagnosis of the disease and consequently improving the prognosis. Furthermore, they could better clarify the mechanisms underlying the progression of the disease and eventually allow the identification of new possible therapeutic approaches. The metabolic profile identified by the considered studies was divided into subgroups to better evaluate the similarities among the class of metabolites.

### 4.1. BCAAs and Kynurenine

Half of the studies taken into consideration in this systematic review have demonstrated a positive correlation between circulating BCAAs (isoleucine, leucine, and valine) and NAFLD/NASH syndromes. The analysis of the adipose tissue demonstrated that obesity and insulin resistance can induce a decrease in the BCAAs intracellularly and an increased concentration in blood of the same aminoacids [11,12,14,15,16]. Several previous studies have suggested that BCAAs are predictors of insulin resistance and cardio-metabolic risk and are directly correlated with the accumulation of lipids in the liver [23,24,25]. In large-scale cohorts, BCAAs have been inversely associated with insulin sensitivity [25] and directly associated with insulin resistance, fasting blood glucose levels, and TG concentrations [24]. Animal studies have shown that a diet rich in BCAAs can cause significant liver damage, oxidative stress, and hepatocyte apoptosis [26]. The mechanisms determining liver damage are not entirely clear. However, Zhang et al. hypothesized that in adipocytes, BCAA activates AMPKα2 (Adenosine monophosphate (AMP)-activated protein kinase) and stimulates lipolysis, increasing plasma free fatty acids (FFA), and therefore the accumulation in the liver. In the liver, BCAA activates mTOR, which inhibits autophagy and the FFA to triglycerides conversion, blocking the hepatic outflow pathway of FFAs and thus intensifying the lipotoxicity of FFAs. Furthermore, the blockade of autophagy increased cell death by apoptosis [26]. An increase in BCAAs metabolism can cause the accumulation of catabolic intermediates and incomplete oxidation of fatty acids and glucose leading to mitochondrial dysfunction of pancreatic B-cells. Acylcarnitine C3 and C5, generated by the catabolism of BCAAs in the liver and skeletal muscle, have been associated with the direct onset of obesity and insulin resistance [14]. In conclusion, it seems that a high concentration of circulating BCAAs may be responsible for the switch from the lean to NAFLD/NASH phenotype [26]. Another metabolite, kynurenine, was found to be increased in subjects with Metabolic Syndrome. An alteration of the metabolic pathway of tryptophan/kynurenine [12,13], with a consequent increase in plasma levels of kynurenine, seems to be associated with apoptosis, pro-oxidant effects, and an increase in inflammation mediated by the arachidonic acid via [27]. 

### 4.2. Carbohydrates

The carbohydrate metabolism was largely impaired in obese individuals, as demonstrated by the study performed by Lovric et al. [18]. From a biological point of view, the increase in glucose concentration in the blood of obese patients can be determined by the overall adipose tissue, by the increase in IR, and the reduced ability of the adipose tissue to store glucose [28,29]. The product of glycolysis, pyruvate, was also found to increase in obese individuals. A possible explanation involves the conversion of pyruvate into lactate, which is the main precursor of gluconeogenesis during anaerobic glycolysis, which is over-regulated among obese individuals. Despite the interesting findings and assumptions of the study, glucose was the only metabolite that had significant associations with all ectopic fat stores. 

### 4.3. Fatty Acids and Derivatives

Reduced levels of phosphatidylcholine (PC) and sphingolipids have been found in NAFLD patients [15]. A study on human adipocyte cultures from healthy obese patients and sick obese patients showed similar results [30]. Phospholipids are substrates necessary for the synthesis of triglycerides in the liver. A study based on an animal model of metabolic syndrome has demonstrated that reduced PC concentrations may be caused by an increase in adipocyte turnover [31]. In fact, it is hypothesized that unique mutations in the Ccna2 promoter of obese mice influence the expression of the Ccna2 gene in adipose tissue, resulting in increased mitotic activity of adipocytes [31]. In obese patients, a high turnover of adipocytes has been highlighted, as well as an association between adipocyte hypertrophy and metabolic complications [32]. This could explain the reduction in PC, and sphingolipid levels in NAFLD patients. The study by Zhou et al. 2016 [20] showed a reduction in SM levels in patients with NASH. According to the study, this alteration appeared to promote cellular stress, mitochondrial dysfunction, and alteration of the insulin-signalling pathway [33], which is closely associated with the etiopathogenesis of the MetS. The obese status was positively correlated with two unsaturated fatty acids (UFA) DGLA and PA as showed by Ni et al. [19]. Both UFAs appeared to be good inflammation markers useful in predicting the risk of developing metabolic syndrome and monitoring the metabolic status of overweight/obese individuals. In particular, a high concentration of PA is related to the augmented activity of stearoyl-CoA desaturase and hepatic de novo lipogenesis [34] which, in turn, leads to an increased synthesis of diacylglycerol (DAG), which contributes to the inflammatory mechanism through the release of arachidonic acid (AA). DGLA represents a pro-inflammatory agent that stimulates the increase in prostaglandins and leukotrienes that is observed in obesity and MetS. In agreement with Ni et al. [19], the study performed by Kurotani K et al. [35] showed that a high percentage of DGLA was associated with high concentrations of C reactive protein, a sensitive marker of inflammation associated with insulin resistance and T2D. Furthermore, the longitudinal study by Ni et al. showed that fasting UFA concentrations were elevated up to 10 years before the onset of the metabolic syndrome [19]. Surgical or dietary interventions in obese patients showed a significant reduction in UFA. UFAs, compared to saturated fatty acids (SFAs), decreased significantly after weight loss interventions, while they increased more significantly in obese subjects with MetS. These results suggest a more important role of UFAs than SFAs in determining the metabolic state of the individual. However, there is contradictory evidence regarding the effects of high SFA intake on the risk of developing type 2 diabetes mellitus [36]. 

### 4.4. Other Metabolites 

The study performed by Männisto et al. [11] showed a reduction in ketone body levels (β-OHB and acetoacetate) in individuals with NASH compared to individuals with NAFLD. This could be justified by the fact that low levels of FFA, characteristic of the patient with NASH, would lead to a reduction in beta-oxidation and consequently to a reduction in circulating ketone bodies. However, a reduction in FFA has not been fully demonstrated. In several studies, the FFA concentration in individuals with NASH was increased compared to healthy controls and there were no differences between NAFLD and NASH [37]. In addition, genetic alterations of the genes ACAT1 and BDH1 that are involved in the regulation of ketolysis were found to be over-expressed in the liver of individuals with NASH. On the one hand, these results may suggest that the reduction in ketone bodies in individuals with NASH is determined by an increased ketolysis necessary to meet the increased need for acetyl-CoA to feed the Krebs cycle [38]. This hypothesis was also supported by the discovery that the levels of citrate, formed in the Krebs cycle from acetyl-CoA [39], were reduced in individuals with NASH. The reduction in the levels of ketone bodies and citrate in patients with NASH associated with an alteration in the hepatic expression of genes involved in ketolysis suggest a mitochondrial dysfunction typical of the patient with NASH compared to the patient with NAFLD. On the other hand, a study in which the patients were treated with a medium-chain fatty acids rich diet, showed an increase in the urinary concentration of metabolites of the Krebs cycle after twelve weeks [40]. These metabolites, and in particular citrate, seemed to be involved in the onset of obesity and related metabolic complications by increasing the synthesis of de novo fatty acids [41].

**Figure 2:**
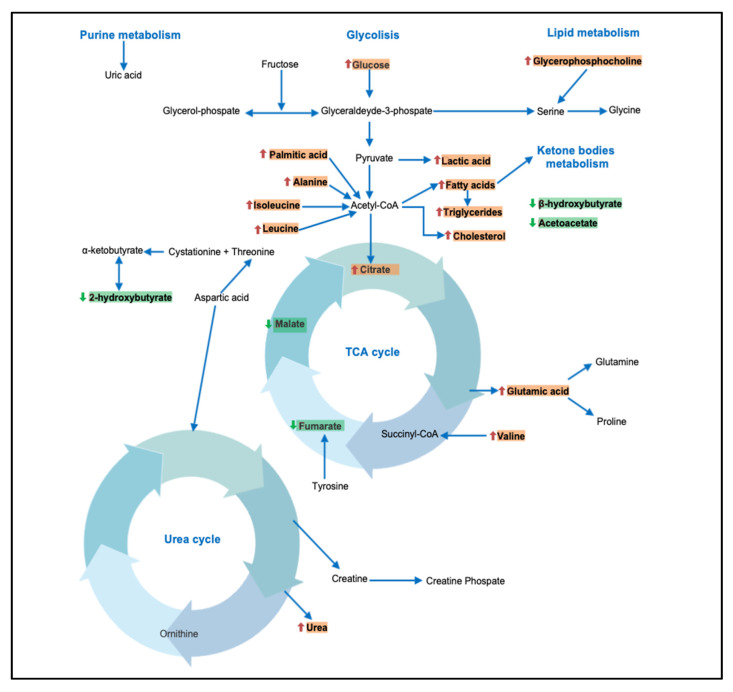
A summary of different pathways potentially involved in NAFLD/NASH. Red arrow: increase in metabolite concentration. Green arrow: decrease in metabolite concentration.

Youm et al. [41] highlighted a potential protective role of β-hydroxybutyrate in inhibiting the NLRP3 inflammasome and reducing the production of IL1β and IL-18 in human monocytes [42]. Several other studies have also shown an alteration in the concentration of other metabolites derived from the Krebs cycle. The study by Sookoian et al. 2016 [13] showed a reduction in citraconic acid and fumaric acid levels in subjects with NAFLD compared to healthy controls. The study by Troisi et al. 2018 [16] showed a reduction in citraconic acid levels in obese subjects compared to healthy controls. Overall, the data indicate an alteration of the Krebs cycle in metabolically ill patients, and this alteration seems to be more marked in subjects characterized by an advanced stage of the disease. In Figure 2, the different pathways and metabolites potentially involved in NAFLD/NASH have been summarized. Furthermore, a brief summary of principal metabolites and their potential role is reported in Table 3.

## 5. Conclusions

Early diagnosis of NAFLD, NASH, and MetS is a relevant topic. This systematic review indicates that the metabolomics approach can identify new biomarkers of these conditions and suggest which are the principal altered metabolic pathways. For the future, there is a need to validate how sensitive these biomarkers are. Further investigations in large and well-characterized cohorts are recommended to gain new insights into the pathomechanisms of these conditions. This will also be useful to improve targeted therapies. Overall, a better understanding of the pathogenesis and the identification of new early biomarkers of MetS will be of value for this highly diffuse condition worldwide, often undiagnosed or diagnosed late.

## Figures and Tables

**Table 1:** Studies excluded after full text review.

Author	Year	Title	Subjects	Reasons of Exclusion
Amer et al.	2017	Consumption of whey in combination with dairy medium-chain fatty acids (MCFAs) may reduce lipid storage due to urinary loss of tricarboxylic acid cycle intermediates and increased rates of MCFAs oxidation	52 abdominally overweight participants	The work analyses the metabolomics profile of patients subjected to different types of diet without distinguishing between patients with metabolic syndrome (or other traits of the latter) and healthy patients
Boden et al.	2015	Excessive caloric intake acutely causes oxidative stress, GLUT4 carbonylation, and insulin resistance in healthy men	3 subjects normal weight (BMI 23.0, 23.9, and 24.9) and 3 overweight (BMI 26.8, 28.7, and 28.1)	The study is not using a metabolomics approach to identify compounds
Chen et al.	2018	Serum metabolomics model and its metabolic characteristics in patients with different syndromes of dyslipidemia based on nuclear magnetic resonance	60 dyslipidemia patients (30 patients with SKYD and 30 patients with PDR) and 20 healthy subjects	Despite the study using a metabolomics approach, exclusively analyses patients with secondary dyslipidemia in SKYD (Spleen and Kidney Yang Deficiency) and PDR (Phlegm-Dampness Retention)
Chen et al.	2019	Trimethylamine N-Oxide Binds and Activates PERK to Promote Metabolic Dysfunction	Mouse model and cell culture	The study is not using a metabolomics approach to identify compounds; The study was not conducted on humans
Grün et al.	2018	High-Density Lipoprotein Reduction Differentially Modulates to Classical and Nonclassical Monocyte Subpopulations in Metabolic Syndrome Patients and in LPS-Stimulated Primary Human Monocytes In Vitro	86 women and men between 20 and 60 years old	The study analyses the differences in monocyte subpopulations in patients with metabolic syndrome using flow cytometry (absence of a metabolomics approach)
Heidenreich et al.	2017	Retinol saturase coordinates liver metabolism by regulating ChREBP activity	Mouse model and cell culture	The study was not conducted on humans
Kempinska et al.	2019	The Association between SOCS11656G>A Polymorphism, Insulin Resistance and Obesity in Nonalcoholic Fatty Liver Disease (NAFLD) Patients	138 patients with features of NAFLD 1000 controls	The study analyses the presence of genetic polymorphisms associated with metabolic syndrome and insulin resistance without using a metabolomics approach for the identification of metabolites
Kozyra et al.	2018	Human hepatic 3D spheroids as a model for steatosis and insulin resistance	Human hepatocyte 3D spheroid cultures	Study conducted on primary human hepatocyte 3D spheroid cultures
Leboucher et al.	2019	The translational regulator FMRP controls lipid and glucose metabolism in mice and humans	25 fragile X patients and 29 sex- and age-matched healthy subjects	The study analyses exclusively people with fragile X syndrome to assess how the absence of specific genes may influence the metabolic homeostasis; No metabolomics approaches were used
Silvestri et al.	2015	Two non-psychoactive cannabinoids reduce intracellular lipid levels and inhibit hepatosteatosis	Mouse model and cell culture	The study was not conducted on humans
Teslovich et al.	2018	Identification of seven novel loci associated with amino acid levels using single-variant and gene-based tests in 8545 Finnish men from the METSIM study	8545 non-diabetic men of mean age 57.3 ± 7.1 years	The study identifies genetic variants related to amino acid alterations in patients with metabolic syndrome
Zimmermann et al.	2010	Alterations in lipid, carbohydrate, and iron metabolism in patients with non-alcoholic steatohepatitis (NASH) and metabolic syndrome	37 patients with metabolic syndrome (25 NASH and 12 non-NASH) 37 controls	The study is not using a metabolomics approach to identify compounds

**Table 2:** Selected Characteristics of Reviewed Studies on the Metabolomics of NAFLD and NASH syndromes.

Author	Years	Sample Size	Biofluid	Technique	Biomarkers Candidates	Results Increase (↑) or Decrease (↓) Biomarker Concentration Compared to the Control Group	Comments
Bhupathiraju et al. [14]	2018	145 participants (aged 45 to 79 years) Control: Vegetarian dietary pattern	Serum	TQD MS/MS	Leucine; Valine; Tyrosine Methionine; Medium (C8–C14) to long-chain (C16–C20) acylcarnitines; Short-chain (C2 and C5) acylcarnitines	BCAAs ↑ Tyrosine ↓ Methionine ↓ Medium (C8–C14) to long-chain (C16–C20) acylcarnitines ↑ Short-chain (C2 and C5) acylcarnitines ↑	Higher scores on the BCAA, aromatic amino acid, and short-chain AC metabolomic pattern were significantly associated with higher fasting insulin and 2-h insulin concentrations
Feldman et al. [15]	2018	183 subjects: 62 obese NAFLD Controls: 69 lean controls 50 obese healthy	Serum	LC-MS/MS	Isoleucine; Leucine; Valine; Sphingolipids (SM); Phosphatidylcholine	In obese NAFLD: Isoleucine ↑ Leucine ↑ Valine ↑ Sphingolipids ↓ Phosphatidylcholine ↓	SM OH C14:1 SM OH C16:1 SM OH C22:2 SM C16:0
Lovric et al. [18]	2018	37 subjects with MetS No Control Group	Plasma	UPLC/MS/MS GC/MS	Cholesterol ester (CE); Triacylglycerol (TAG); Phosphatidylcholine (PC); Glucose; Lysophosphatidylcholine (LPC); Sphingomyelin (SM); DAG (16:0/18:2) *; PE (16:0/18:1) **; PI (18:0/20:4) ***	CE 18:2 ↓ CE 20:3 ↑ DAG (16:0/18:2) ↑ SM (18:0) ↑ PC (38:3) ↑ PE (16:0/18:1) ↑ PI (18:0/20:4) ↑ TAG (50:4) ↓ TAGs containing FA with C48 to C50 ↑ TAGs containing FA with C52 to C58 ↓ Glucose ↑	Ectopic fat (myocardial, epicardial, pericardial, and liver) was measured in non-diabetic male subjects with NAFLD Trend of plasma metabolites correlated with the increase in fat levels in ectopic fat deposits
Männisto et al. [11]	2014	76 obese individuals: 32 with normal liver, 19 with simple steatosis 25 with NASH Controls: 32 with normal liver	Serum	^1^H-NMR	Alanine; Histidine; Isoleucine; Leucine; Phenylalanine; Tyrosine; Valine; Citrate; β-hydroxybutyrte (β-HB); Acetoacetate	Alanine ↑ (NASH) Histidine ↑ (NASH) Isoleucine ↑ (NASH) Leucine ↑ (NASH) Phenylalanine ↑ (NASH) Valine ↑ (NASH) Citrate ↑ (NASH) β-HB ↓ (NASH) Acetoacetate ↓ (NASH)	β-HB decreases in the NASH compared to NAFLD Low levels of ketone bodies were associated with liver cell injury (ballooning)
Masarone et al. [17]	2021	69 Controls 78 NAFLD 23 NASH 15 NASH-cirrhosis	Serum	GC-MS	Glycocholic acid; Taurocholic acid; Phenylalanine; BCAAs; Glutathione	Glycocholic acid ↑ (NASH) Taurocholic acid ↑ (NASH) Phenylalanine ↑ (NAFLD and NASH) BCAAs ↑ (NAFLD and NASH and NASH-cirrhosis) Glutathione ↓ (NAFLD and NASH)	
Ni et al. [19]	2015	132 healthy subjects with normal weight (NW). 107 overweight/obese metabolically healthy (HO) 73 overweight/obese diabetic individuals (UO)	Serum	UPLC-QTOF-MS	Palmitoleic acid (PA) Dihomo-γ-linolenic acid (DGLA)	PA ↑ DGLA ↑ (UO)	Palmitoleic acid increase in UO subjects compared to HO subjects
Sookoian et al. [13]	2016	32 patients with NAFLD 16 healthy controls	Serum	HPLC-MS	Alanine:pyruvate ratio; Kynurenine; Methionine; Taurine; Glucose; Glucose-6-phosphate; Lactic acid; Citraconic acid; Fumaric acid; Methyladenosine; N2, dimethylguanosine	Alanine:pyruvate ratio ↑ Kynurenine ↑ Methionine ↑ Taurine ↓ Glucose ↑ Glucose-6-phosphate ↓ Lactic acid ↑ Citraconic acid ↓ Fumaric acid ↓ Methyladenosine ↑ N2, dimethylguanosine ↑	
Stechemesser et al. [12]	2016	53 healthy controls 54 MetS without hyperferritinemia (MetS-Fe) 56 MetS with hyperferritinemia (MetS+Fe)	Serum	LC-MS/MS	Alanine; Citrulline; Glutamate; Kynurenine; Leucine; Sarcosine; Valine; Long-chain PCs;	Alanine ↑ Citrulline ↑ (MetS+Fe) Glutamate ↑ Kynurenine ↑ Leucine ↑ Sarcosine ↑ (MetS+Fe) Valine ↑ Long-chain PCs ↑ (MetS+Fe)	PC 40:2 PC 40:3 PC 40:4 PC 42:1
Troisi et al. [16]	2019	23 obese patients (15 with hepatic steatosis (St+); 8 without hepatic steatosis (St–); 10 with MetS; 13 without MetS) 18 normal weight healthy controls	Saliva	GC-MS	Isoleucine; Urea; Aconitic acid; Erythrose; Gluconic acid; Maltose; N-acetylgalactosamine; Lauric acid; Palmitic acid; Citraconic acid; Hydroxybutyric acid; Malic acid; Methylmalonic acid; Myristic acid	Isoleucine ↑ Urea ↑ Aconitic acid ↑ (Obese with MetS) Erythrose ↓ (Obese with MetS) Gluconic acid ↑ Maltose ↓ (with NAFLD) N-acetylgalactosamine ↑ Lauric acid ↓ (with NAFLD) Palmitic acid ↑ Citraconic acid ↓ Hydroxybutyric acid ↓ (without NAFLD) Malic acid ↓ (without NAFLD) Methylmalonic acid ↓ (Obese with MetS) Myristic acid ↑	
Zhong et al. [21]	2019	22 livers with NAFLD or NASH 50 normal liver	Liver Samples	LC-MS/MS	all-trans-retinoic acid (atRA); 13-cisRA; 4-oxo-arRA; all-trans-retinyl palmitate-d4 (RP)	RP ↓ (NASH) atRA ↓ (NASH) 13-cisRA ↓ 4-oxo-arRA ↓	4-oxo-arRA decrease in both NASH and NAFLD
Zhou et al. [20]	2016	117 with NAFLD 69 NASH Control group	Plasma	UPLC-MS	Lysophosphatidylcholine 16:0 (LysoPC 16:0); Sphingomyelin; Unsaturated triacylglycerols (UnTGs)	LysoPC 16:0 ↓ (NASH) Sphingomyelin ↓ (NASH) UnTGs ↑(NASH)	

* 1-palmitoyl-3-linoleoyl-glycerol (16:0/18:2); ** 1-palmitoyl-2-oleoyl-sn-glycero-3 phosphoethanolamine; and *** 1-stearoyl-2-arachidonoyl-sn-glycero-3-phosphoinositol.

**Table 3:** Brief tabular summary of principal metabolites and their potential role.

Metabolites	Biofluids	Potential Effects
BCCAs *(isoleucine, leucine, and valine)*	Serum	Potential predictors of insulin resistance, fasting blood glucose level, and TG concentration
Kynurenine	Serum	The increase of Kynurenine associated with apoptosis and pro-oxidant effect (inflammation)
Glucose	Serum	The hyperglycemia condition is one of the main causes in the development of insulin resistance
Lactate	Serum	Precursor of gluconeogenesis during anaerobic glycolysis. Pathway overexpressed in the metabolic syndrome
Phosphatidylcoline *(PC)*	Serum	The reduction in CP levels appears to be caused by an increase in the turnover and size of adipocytes
Sphingolipids *(sphingomyelin, ceramides)*	Serum	The alteration of the sphingolipids seems to promote cellular stress, mitochondrial dysfunction, and alteration of the insulin-signalling pathway
DGLA *(dihomo-gamma-linolenic acid)*	Serum	Obese status was positively correlated with DGLA. DGLA is a good inflammation marker, useful in monitoring the metabolic status of overweight/obese individuals
Palmitoleic acid *(PA)*	Serum	An increase in PA is associated with an increase in de novo lipogenesis, which leads to an increase in DAG synthesis
Ketone bodies	Serum	A reduction in β-hydroxybutyrate levels seems to inhibit the NLRP3 inflammasome and reduce the production of IL1beta and IL-18.

## Data Availability

All data is available within the article.
